# A Review of Evidence for Surgical Closure Versus Medical Therapy Alone in Preventing Recurrent Stroke in the Presence of a Patent Foramen Ovale

**DOI:** 10.7759/cureus.96983

**Published:** 2025-11-16

**Authors:** William Cooke

**Affiliations:** 1 Department of Medicine and Surgery, University of Liverpool, Liverpool, GBR

**Keywords:** anticoagulation, cryptogenic stroke, dapt, dual antiplatelet therapy, patent foramen ovale, percutaneous atrial septal defect closure, pfo

## Abstract

Presented is a review of the evidence for surgical closure of a patent foramen ovale (PFO) vs. medical therapy alone in the prevention of recurrent stroke in those who have already suffered a cryptogenic stroke. Several trials that made this comparison were identified. Their methodologies and results were analysed in an attempt to make a unifying conclusion about whether surgical closure is beneficial and for which groups of patients.

A literature search identified six significant trials conducted over the last two decades, which recruited a total of 3,750 participants aged 60 years or younger. Their methodologies, including inclusion and exclusion criteria, device choices, endpoint definitions and published results, were evaluated and compared.

Early trials were unable to demonstrate a benefit from closure. Later trials found a significant benefit to closure. The later trials refined the inclusion and exclusion criteria to recruit more at-risk participants, specifically those with large atrial septal aneurysms (ASAs) and large shunts across the atrial septum. There were procedural concerns with all trials to varying degrees. Concerns included the open nature of the trials, vague inclusion/exclusion criteria, participants treated outside of their assigned designation, lack of adequate power and mid-trial changes to the defined outcome measures.

It appears there is evidence in favour of PFO closure in the prevention of recurrent stroke in young patients with large ASAs and large shunts. PFO closure was associated with significantly higher rates of atrial fibrillation and serious procedure-related complications, so the relative risk and reward must be determined on a patient-to-patient basis. A decision should be made only after a thorough investigation, which is to include bubble echocardiography, long-term ECG/Holter monitoring and exclusion of non-PFO-related index strokes via a comprehensive search for alternate aetiologies.

## Introduction and background

A foramen ovale is a connection between the right and left atrium within the heart. This is present during foetal development to allow circulating blood to bypass the pulmonary vasculature. The foramen ovale usually closes soon after birth, but in approximately 25% of people, a patent foramen ovale (PFO) persists into adulthood [1].

In individuals presenting with cryptogenic stroke, that is, a stroke without an identified cause, a PFO is present in approximately 40% of patients, suggesting PFO as a significant risk factor for the development of cryptogenic stroke and transient ischemic attack (TIA) [2]. The stroke or TIA is considered likely to be caused by the PFO's presence if there is no reasonable competing aetiology, which typically means younger patients without a significant history of cardiovascular disease, haematological conditions, known arrhythmias, and other risk factors. Consequently, studies that look at PFO-related cryptogenic strokes typically look at younger and healthier populations than would otherwise be expected in a population who have experienced a cerebrovascular event.

**Figure 1:**
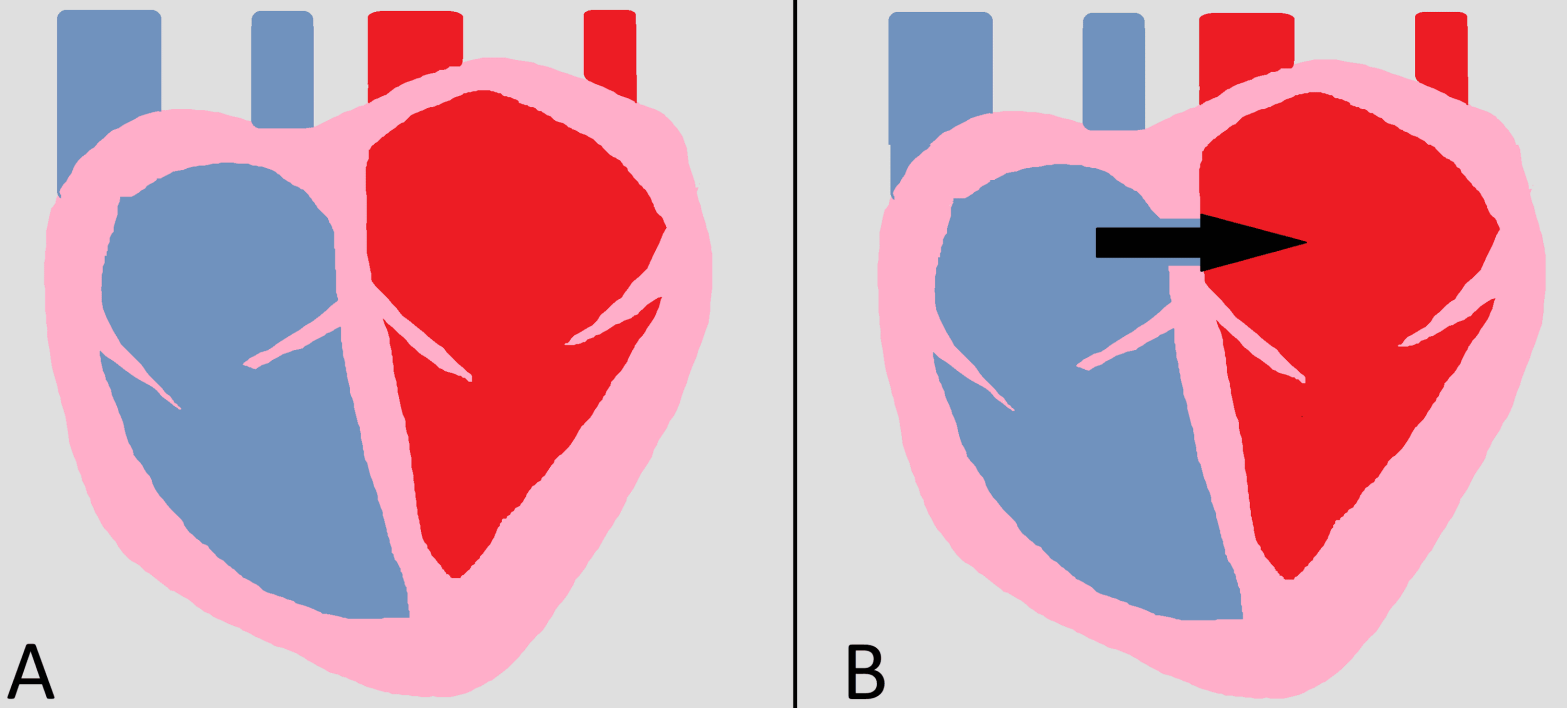
A simple drawing showing (A) a normal human heart and (B) a heart with a patent foramen ovale allowing a connection between the right and left atrium Image credit: This is an original image created by author William Cooke

The likely mechanism for how PFO leads to cryptogenic stroke involves the formation of a venous embolism, which would typically become lodged in the pulmonary vasculature but instead, through right-to-left shunting across the atrial wall, ends up in the systemic arterial supply, leading to embolic stroke, a phenomenon known as paradoxical embolism [3]. A simple drawing showing this is seen in Figure 1. Other proposed mechanisms involve the development of thromboses within the PFO itself, resulting from turbulent blood flow [4].

Various studies have assessed the characteristics of the PFO and associated findings to identify individuals at particular risk of cryptogenic stroke [5,6]. PFO size can be determined on a transoesophageal echocardiogram (TEE) and the degree of right-to-left shunting with a bubble contrast echocardiogram, a 'bubble echo'. The bubble echo measures the number of microbubbles shunted across the atrial septum and can be used to quantify the possible clinical significance of the PFO. There is evidence to suggest that a larger PFO increases the risk of cryptogenic stroke [6]. An atrial septal aneurysm is a bulge of the atrial septum that is often found alongside a PFO. The presence of ASA and PFO has been shown to increase the risk of cryptogenic stroke [7].

Due to the high prevalence of PFO in the population and the high burden of disease experienced by many of these younger and healthier individuals suffering from cryptogenic stroke, there has been much thought on how best to prevent recurrent stroke. Prevention strategies have largely been categorised into three main areas: 1) antiplatelet therapy, e.g., aspirin/acetylsalicylic acid, to help prevent thrombus formation; 2) anticoagulation therapy, e.g., warfarin or apixaban, to prevent thrombus formation; and 3) surgical closure of the PFO with an intracardiac device. This prevents shunting and therefore prevents paradoxical embolism.

Over the last two decades, several large trials have been conducted to determine the relative benefits of these treatment options. This paper aimed to find the relevant trials, evaluate them and see if there is substantial enough evidence for the surgical closure of PFO vs. medical therapy alone in the prevention of recurrent stroke in those with a history of cryptogenic stroke.

## Review

Methods

**Figure 2:**
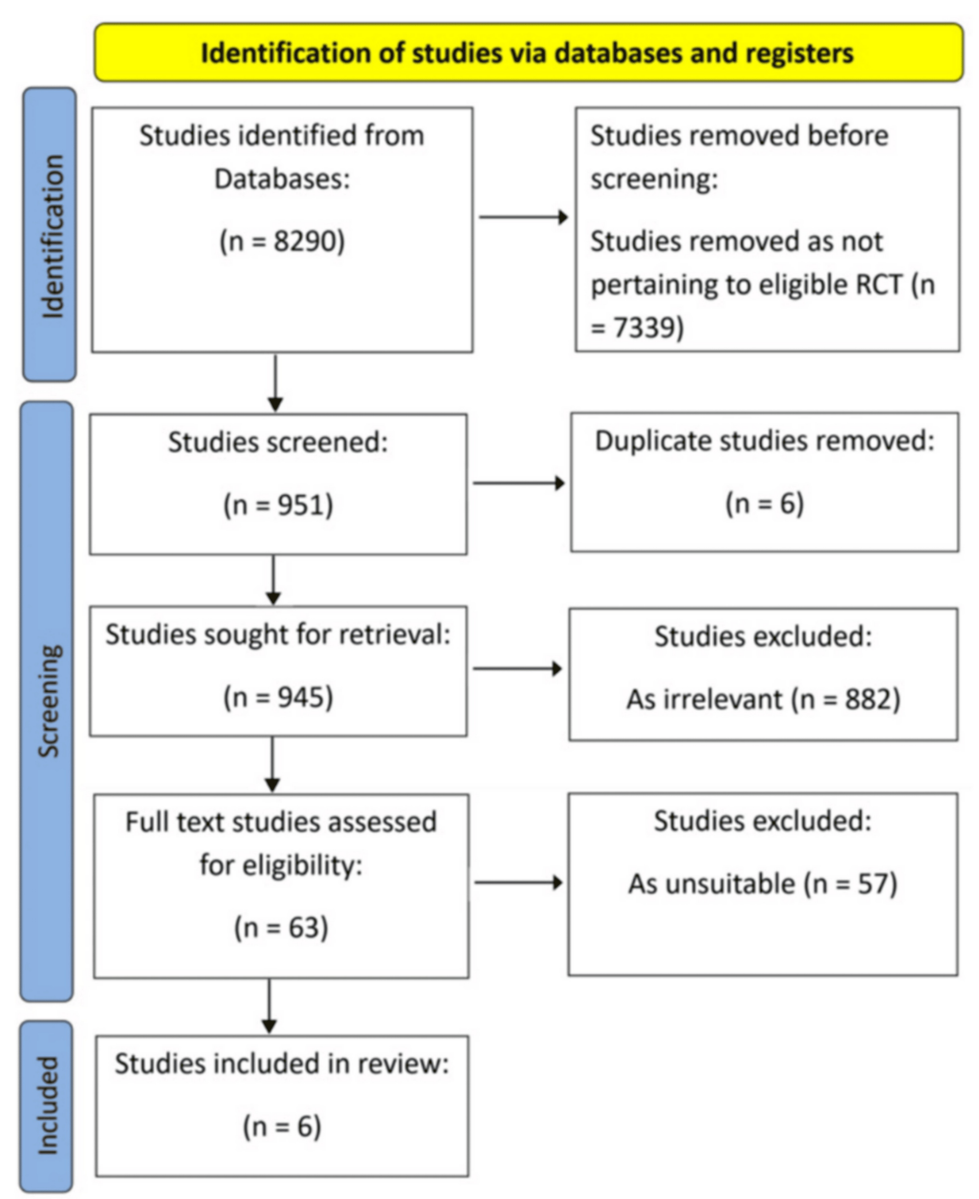
Results and exclusions following the database search

A literature search was conducted on PubMed and Scopus using search terms of ‘Stroke’, ‘Cryptogenic’, ‘Patent foramen ovale’, ‘PFO’, ‘TIA’, ‘device’, ‘closure’, ‘antiplatelet’ and ‘anticoagulation’. The results, as shown in Figure 2, were systematically filtered to find the original large-scale trials. After identifying the relevant trials, they were critically appraised and reviewed to identify any areas where consensus could be drawn. Where possible, it was hoped to collate data from different trials.

The literature search identified six relevant trials. The findings of each trial have been summarised in the following tables to identify the strengths and weaknesses of each.

CLOSURE I (2012)

The results indicate that there is no benefit to closure vs. medical therapy at a 95% confidence interval. The inclusion of TIA as an endpoint is controversial, as it is a less definitive measure than stroke or death [8]. They felt it necessary to include TIA as the participant numbers to reach a significant conclusion would be prohibitively high when relying on stroke alone.

According to the protocol, participants were to be excluded if they had other risk factors that would lead to a suspicion that the stroke was caused by something other than an R-to-L shunt through a PFO. This was undertaken at a time when more reproducible methods for identifying PFO as the cause of cryptogenic stroke had not yet been established, such as the risk of paradoxical embolism (RoPE) or PFO-Associated Stroke Causal Likelihood scores, which may have led to some questionable inclusions [9].

At randomisation, among other risk factors, 31% of participants had hypertension and 44% had hyperlipidaemia. This casts doubt on what proportion of the initial stroke or TIAs were related to PFOs after all. The protocol also allows for the inclusion of patients presenting with lacunar infarcts, which are unlikely to be PFO-related.

Of the 909 participants, 5.5% of the closure group progressed to the composite endpoint, compared to 6.8% in the medical therapy group. Of the 52 participants who had a recurrent stroke or TIA within two years, 80.7% of them had possible aetiologies that were not PFO related, including atheroma, vasculitis and atrial fibrillation (AF).

Another limitation of this study is the use of only one permitted device by the study sponsors, the StarFlex device (NMT Medical, Boston, Massachusetts). Subsequent studies have shown it to be a less effective option than other devices and in this study produced several periprocedural complications, including AF in 6% of cases [10]. Of the 447 allocated to the closure group, only 315 were shown at six months to have effectively closed the PFO. Four patients were found to have a thrombus formed in the left atrium at six months, and the device itself may have contributed to this. The details of the trial are summarised in Table 1.

**Table 1:** CLOSURE I (2012) details TIA: transient ischemic attack; PFO: patent foramen ovale Source: [11]

Study characteristic	Description
Dates	2003-2008
Location	USA and Canada
Participants	909
Type	A multicentre, randomised, open-label trial in the USA and Canada
Age	18-60
Included	Participants had suffered a stroke or TIA within 6 months and had a PFO
Arm 1	Percutaneous closure of PFO with the StarFlex device and antiplatelet therapy (81 or 325 mg of aspirin)
Arm 2	Medical therapy alone with warfarin and/or aspirin
Endpoint	Participants were followed up over 2 years, checking for a composite outcome of subsequent stroke, TIA or death

PC trial (2013)

The results indicate that there is no benefit to closure of the PFO vs. medicinal therapy alone. The results revealed a nonsignificant benefit to closure, especially in those under 45 (hazard ratio, HR: 0.16 (0.02-1.31); p = 0.10) and those without an atrial septal aneurysm (ASA; HR: 0.32 (0.09-1.18); p = 0.09,) but neither could be established to a 95% confidence interval.

Of those fitted with a closure device, 23% were not assessed via bubble echo at six months to determine the level of success. Of the 148 individuals assessed in this manner, 95.9% were considered to have achieved a successful closure.

There were some potentially significant differences between the two study arms. Those in the closure arm were more likely to be women (54.9% vs. 45.7%), have a family history of cerebrovascular event (26% vs. 19%) and have medium or large shunts (70.3% vs. 60.9%). Of the 210 assigned to have medical therapy only, 28 (>13%) ultimately had PFO closure anyway.

This study raises the question of anticoagulation vs. antiplatelet therapy alongside the surgical PFO closure. At 12-month follow-up, only 2.6% of those in the closure group were taking an anticoagulant compared to 22.2% in the medical therapy group.

The inclusion and exclusion criteria were similar to those of the CLOSURE I study. This study also had too few participants enrolled and was not adequately powered to answer the research question. The details of the trial are summarised in Table 2.

**Table 2:** PC trial (2013) details TIA: transient ischemic attack; PFO: patent foramen ovale; AF: atrial fibrillation Source: [12]

Study characteristic	Description
Dates	2000-2009
Location	Europe, Canada, Brazil and Australia
Participants	414
Type	A multicenter, superiority trial in Europe, Canada, Brazil and Australia
Age	18-60
Included	Participants had a history of PFO and occurrence of stroke, TIA or peripheral embolism. In this case, the TIA and peripheral embolism required clinical and radiological confirmation
Arm 1	Amplatzer PFO occlusion + acetylsalicylic acid (aspirin) + ticlopidine or clopidogrel
Arm 2	Antithrombotic only. Antiplatelet or anticoagulation at the discretion of the physician
Endpoint	Participants were followed up for an average of 4.1 years looking for the primary endpoint of a composite of death, stroke, TIA or peripheral embolism. Secondary endpoints included the occurrence of new AF

RESPECT trial (2013)

The results indicate that there is no benefit to closure vs. medical therapy on an intention-to-treat basis. On a per-protocol and as-treated basis, however, closure was significantly better at reducing recurrent stroke than medical therapy alone.

The results suggest closure is most beneficial with larger shunts and ASAs. They assessed and recorded infarct size, and these were considerably smaller in the closure group, suggesting a prevention of more clinically significant strokes [13]. The rates of successful closure were better (93.5%) at six months than with the StarFlex device, and only 3% experienced AF. This was not significantly more than the nonclosure group.

Although unable to confirm a benefit on an intention-to-treat basis, this study suggested that a possibly safer and more effective device was available and that, when used in more at-risk patients, could provide a benefit. The trial results were initially reported in 2013, but some outcome measures were followed up until 2015, and this was reported separately in 2017. Where possible, the longer-term data were used [14]. The details of the trial are summarised in Table 3.

**Table 3:** RESPECT trial (2013) details PFO: patent foramen ovale Source: [15]

Study characteristic	Description
Dates	2003-2011
Location	USA and Canada
Participants	980
Type	Prospective, multicentre, randomised, event-driven trial
Age	18-60
Included	Participants had a PFO and a 'cryptogenic' stroke within the last 270 days
Arm 1	Amplatzer PCO occluder (+81 to 325 mg aspirin + clopidogrel for 1 month, aspirin monotherapy for 5 months, then antiplatelet therapy at the discretion of the investigator)
Arm 2	Aspirin, warfarin, clopidogrel or aspirin + dipyridamole
Endpoint	Participants were followed up for an average of 2.6 ± 2 years with composite endpoints of fatal stroke, nonfatal stroke and early death (within 45 days of randomisation or 30 days of device insertion). The trial would continue until 25 primary endpoints occurred

CLOSE trial (2017)

The results indicate a significant improvement in HR in the closure group compared to antiplatelet therapy. It found no significant benefit to anticoagulation compared to antiplatelet therapy. This was the first major trial to standardise post-procedure anticoagulation, allowing for fewer confounding variables in that arm. It also stringently separated the anticoagulation vs. antiplatelet groups rather than giving practitioners discretion as with CLOSURE, RESPECT and PC trials.

It had more stringent recruitment criteria and included the use of the Trial of Org 10172 in Acute Stroke Treatment (TOAST) classification to confirm the type of stroke and the RoPE score to aid determination of PFO as the cause of the stroke [16]. Pleasingly, rates of hypertension and hypercholesterolaemia were significantly lower than in previous studies. Crucially, it included only patients with cryptogenic stroke attributed to PFO and with large shunts or ASA: this is the more at-risk group, which would better study the target population but also allow higher numbers reaching the endpoint and therefore a better power to the study.

It found AF in 4.6% of the closure group, which is significantly more than that in the medical therapy group. The trial used 11 different devices, with 50% being Amplatzer PFO occluders (Abbott Laboratories, Abbott Park, Illinois). It would have been interesting to see if AF rates or other complications varied by device type, e.g., were all AF patients fitted with a StarFlex device? Unfortunately, the published results did not allow for this subgroup analysis. The details of the trial are summarised in Table 4.

**Table 4:** CLOSE trial (2017) details PFO: patent foramen ovale Source: [17]

Study characteristic	Description
Dates	2007-2016
Location	France and Germany
Participants	663
Type	Investigator-initiated multicentre, randomised, open-label, superiority trial
Age	18-60
Included	Participants had a PFO and a history of 'cryptogenic' stroke
Arm 1	Closure + dual antiplatelet therapy of 75 mg aspirin + 75 mg clopidogrel daily
Arm 2	Anticoagulation
Arm 3	Antiplatelet
Endpoint	The study aimed to find a benefit to closure of PFO vs. antiplatelet therapy in the prevention of recurrent stroke and separately to find a benefit of antiplatelet therapy over anticoagulation

Gore REDUCE trial (2017)

The results indicate a significant benefit to closure in reducing the incidence of clinical stroke. Nonsignificant reduction in silent stroke, this was demonstrated in the intention-to-treat, per-protocol and as-treated analysis. Rates of AF were 6.6% in the closure group compared with 0.4% in those receiving antiplatelet medication. Worryingly, 41% had not resolved within two weeks, and one AF patient went on to have a recurrent stroke. It is worth noting that overall, serious adverse events were higher in the medical group than the closure group at 23.1% compared with 27.8%.

Although the results of this trial were promising, there were a few concerns with the trial's conduct. The protocol initially permitted one device (Helix Septal Occluder, Gore Medical, Newark, Delaware), but a second (Cardioform Septal Occluder, Gore Medical) was added partway through. This skewed the allocation numbers. The second primary endpoint was also a mid-trial inclusion. The publishers benefited from seeing the results of previous trials and added the broader secondary endpoint due to low rates of participants reaching the endpoint in other trials.

Generally, the inclusion and exclusion criteria were well-defined, especially in excluding individuals with other potential causes of the index stroke unrelated to PFO. This included angiography to detect atherosclerosis and exclusion of lacunar infarcts. There was no stratification for ASA before randomisation, unfortunately. We also saw higher levels of dropouts in the medical therapy groups, as with other trials (9% vs. 15%). The details of the trial are summarised in Table 5.

**Table 5:** Gore REDUCE trial (2017) details PFO: patent foramen ovale Source: [18]

Study characteristic	Description
Dates	2008-2015
Location	Scandinavia, UK, USA and Canada
Participants	664
Type	Multinational, prospective, randomised, controlled, open-label trial
Age	18-60
Included	Participants had a PFO and a 'cryptogenic' stroke
Arm 1	Closure + antiplatelet (clopidogrel 300 mg periprocedurally followed by 75 mg for 3 days and then ongoing treatment at practitioner discretion)
Arm 2	Antiplatelet only (practitioner discretion to choose between aspirin only, aspirin and dipyridamole in combination, or clopidogrel only)
Endpoint	Clinical evidence of stroke and a combination of clinical evidence of stroke or evidence on imaging at 24 months of a silent stroke

DEFENSE-PFO trial (2018)

The result indicates a significant benefit to closure vs. medical therapy. This trial only included patients with higher risk, larger PFOs, ASAs and atrial hypermobility. It included an older cohort (mean: 51.8 years old). There were good inclusion and exclusion criteria, including angiography to exclude significant stenosis and Holter monitoring to exclude paroxysmal AF. Unfortunately, randomisation was not stratified, and the medical therapy group had higher proportions of ASAs, hypertension, diabetes, current smokers and hypercholesterolaemia, which may reduce the reliability of the result. The trial was underpowered due to early termination, citing safety/ethical concerns following the release of other trials suggesting a benefit to closure. AF occurred in two of the closure groups. The details of the trial are summarised in Table 6.

**Table 6:** DEFENSE-PFO trial (2018) details PFO: patent foramen ovale Source: [19]

Study characteristic	Description
Dates	2011-2017
Location	South Korea
Participants	120
Type	Randomised, controlled, open-label trial
Age	18-60
Included	Participants had a high-risk PFO and a 'cryptogenic' stroke
Arm 1	Closure Amplatzer PFO Occluder + (aspirin 100 mg/day in combination with clopidogrel 75 mg/day) for at least 6 months after the procedure, but practitioners are given discretion to change
Arm 2	Antiplatelet - aspirin, aspirin in combination with clopidogrel at a dose of 75 mg/day, or aspirin in combination with cilostazol at a dose of 200 mg/day. Warfarin was used to maintain the target international normalised ratio of 2.0:3.0
Endpoint	The defined outcome measures were stroke, death or thrombolysis in myocardial infarction-defined major bleeding during 2 years of follow-up

Discussion

**Figure 3:**
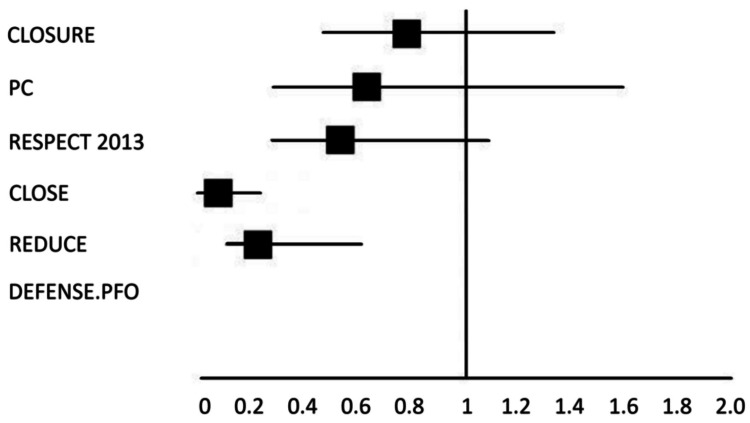
Hazard ratios for primary endpoint across the trials Coprimary endpoint 1 is used for the REDUCE trial The DEFENSE-PFO trial was insufficiently powered to provide a hazard ratio Source: [11,12,15,17-19]

All the trials suggested a benefit to device closure over medical therapy, but only the latter trials could prove it to a 95% confidence interval. The HRs are shown in Figure 3, with the later trials suggesting a significant benefit to closure. The DEFENSE-PFO trial had zero subjects in the closure arm reach the endpoint of the primary outcome measure vs. six in the control arm, but unfortunately, it was not sufficiently powered to calculate an HR.

All the trials found that the rates of progression to the primary endpoints were lower than expected. Consequently, some of the trials were not sufficiently powered to make a significant conclusion. Some deviated from protocol and altered assigned outcome measures mid-trial in light of this.

Earlier trials typically had wider inclusion criteria, and this raised the possibility that many of the strokes before and after admission to the trial may not have been related to the PFO; therefore, these participants should have been excluded. Some had high proportions of patients with other cardiovascular risk factors, e.g., diabetes, hypertension, smoking and family history of cardiovascular disease. Better trials incorporated RoPE scores to help include only PFO-related index strokes or stratify participants between the groups according to their score. Better trials also used the TOAST classification to exclude participants whose index stroke was of a type which is substantially more likely to be caused by another factor, e.g., small lacunar infarcts.

Later trials recognised the increased risk of recurrent stroke posed by larger PFOs and ASAs and sought to preferentially include these subjects in the study, as this group would be expected to have higher rates of recurrent stroke and, therefore, provide a more powerful study.

**Figure 4:**
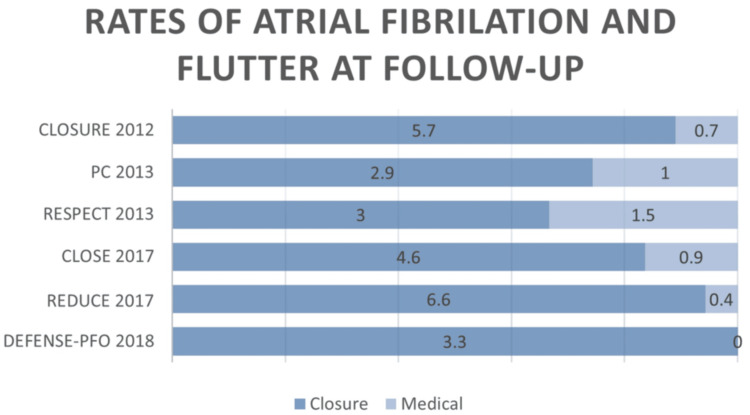
Proportion or AF occurring in the closure group vs. medical group AF: atrial fibrillation Source: [11,12,15,17-19]

All studies identified AF and atrial flutter as complications related to device implantation, the rates of which can be seen in Figure 4 and Table 7. Fortunately, most of these were only temporary. The only studies that offered multiple devices for comparison of AF rates or percentage of successful closure did not allow for easy comparison between different devices. The studies that included only Amplatzer devices seemed to have lower rates of AF and flutter than those that allowed for others, suggesting this may be a safer device and, on average, provided more successful closure of PFO on echo studies. It could be suggested that some participants who experienced AF may have been existing sufferers of paroxysmal AF. The PC trial and RESPECT advised a 24-hour ECG or Holter monitoring when suspecting AF, but it was not a requirement for all participants. Only the Defence-PFO trial and the CLOSE trial made this requirement of all participants to rule out preexisting paroxysmal AF with any confidence.

**Table 7:** AF and atrial flutter rates and rates of effective closure of PFO AF: atrial fibrillation; PFO: patent foramen ovale Source: [11,12,15,17-19]

Trials	Device	Effective closure of PFO	Rates of Afib and flutter device/medical
CLOSURE 2012	StarFlex	86.1	5.7/0.7
PC 2013	Amplatzer PFO occluder	95.9	2.9/1.00
RESPECT 2013	Amplatzer PFO occluder	93.5	3.0/1.5
CLOSE 2017	Amplatzer PFO occluder, StarFlex +9 others	93	4.6/0.9
REDUCE 2017	Helex Septal Occluder or Cardioform Septal Occluder	94.5	6.6/0.4
DEFENSE-PFO 2018	Amplatzer PFO occluder	100	3.3/0.0

In addition to the higher rates of supraventricular arrhythmias, the closure groups were more at risk of procedure-related complications, which included pericardial tamponade, pulmonary embolism and thrombus formation. Unfortunately, comparison and collation between trials were made difficult by the inconsistencies in outcome measures. This was especially true of the primary outcome measures, some of which included stroke, TIA, death of all causes, neurological causes as well as varying requirements to confirm stroke or TIA with imaging or not. There were also differing measures for determining the success of PFO closure, as well as determining what constitutes a large PFO and a large degree of shunting. For example, the RESPECT trial recorded a grade III shunt as >20 microbubbles, REDUCE defined its ‘large’ category as >25, and CLOSE used >30 microbubbles.

All studies suffered from subjects being offered an option other than the one they were assigned. This was especially true of people assigned medical therapy who went on to have surgical closure, suggesting that perhaps higher risk patients were offered device closure regardless of assignment. Further per-protocol analysis was conducted where possible, which drew firmer conclusions but introduced an element of selection bias as these higher risk individuals would be discounted.

There was a lot of variability and physician discretion in the choice of medical therapy to be given. Only the CLOSE trial attempted to distinguish between antiplatelet and anticoagulation therapies. It found a nonsignificant benefit to anticoagulation over antiplatelet therapy, though anticoagulants are associated with higher rates of haemorrhagic complications.

Recent meta-analyses have largely drawn similar conclusions that device closure is superior to medical therapy and that rates of AF are significantly higher in those fitted with a device [20]. Globally, professional bodies have begun to update their guidance to reflect the new evidence from these trials. In 2020, the American Academy of Neurology updated its guidance to recommend PFO closure in patients younger than 60 with a PFO and embolic-appearing infarct and no other mechanism of stroke [21]. In the UK, the clinical commissioning policy was updated in 2019 to support closure in those ‘aged around 60 or younger’ in whom stroke had been confirmed with imaging and other potential risk factors had been ruled out [22].

## Conclusions

In young patients with cryptogenic stroke, especially those with large PFOs and ASAs, there is a benefit to device closure vs. medical therapy alone. There is an increased risk of AF and atrial flutter in those fitted with a device. It is important that when considering fitting a device to close a PFO following a cryptogenic stroke, thorough investigations take place to determine other potential causes for the stroke. The use of RoPE scores or similar may form part of this, but so should long-term ECG monitoring for potential arrhythmias and imaging to rule out lacunar infarcts. Amplatzer devices may be a safer and more reliable device than others, especially the earlier StarFlex device, which has been discontinued.

We have very little data on how the conclusions drawn from these trials can be applied to individuals over 60 and under 18 years of age. Therefore, a trial in these age groups may be worthwhile, although the participant numbers that would likely be required may be prohibitive.
